# General Notes

**Published:** 1893-11

**Authors:** 


### GENERAL NOTES.

The lack of suitable foods for convalescents from severe illness, and in the treatment of typhoid and other low fevers is often felt by the practical physician. Milk while of very great use often cannot be taken, and often causes trouble on account of the indigestibility of the casein. The various beef extracts are more stimulating than nourishing, and the majority of the prepared foods offered are either unpleasant to the taste, or difficult of digestion, and unsuited to the needs of the case.

In such cases Malted Milk will form a very welcome addition to the dietary of the sick room. The basis of this food is pure, fresh, sterilized milk, in which the casein is rendered digestible by the action of the plant pepsin produced by a special method of malting the cereals originated by the manufacturers. It is pleasant to the taste, simply prepared by dissolving in water, no cooking required, and will be retained and assimilated in many cases when all other foods fail.

This is what Dr. E. J. Worst, of Ashland, Ohio, agrees to do for all our readers. He is the discoverer of the famous Australian Electro Pill remedy that acts similar to electricity upon the nervous system in curing kidney, liver and stomach trouble and all sympathetic diseases. This is a very generous act, and we hope that all our readers will avail themselves of this generous offer. All you need to do is to write him a postal card and name Hall's Journal of Health, and he will mail you seven days' trial treatment free. Address above.

Southern Pines, Moore County, North Carolina.—Is a new winter health resort just coming into prominent notice. It is located in the high dry long leaf pine sand hills, amid the tar, pitch and turpentine district.

Thousands of Northern invalids have visited the place and many remarkable cures have been effected. Prominent physicians have visited the place for investigation and without a single exception say, it is the best in the United States, and we are specially requested by Mr. John T. Patrick, Commissioner of Immigration for the Southern states, to invite physicians of the Northern and Western states to visit the place and investigate in the interest of their patients.

Any physician desiring information can address Mr. Patrick, at Pine Bluff, N. C., or the Recreation Bureau of this journal will gladly furnish any desired information.

It is surprising what a popularity the Royal Baking Powder has attained in the last decade of years. New so-called baking powders spring up like mushrooms; but they die as soon, why ? Because the Royal is the only pure and genuine powder on the market.

We welcome once more to our advertising department that wonderful medicinal property, Scott's Emulsion. There is nothing on the market that can compete with it. It should be a visitor in every home.

Dr. Ross, of Grand Rapids, Mich., announces in this number of the Journal what he can do, and the doctor is one of the leading physicians of the West. What he says he can do, believe him.

